# Determinants of Well-Being Among Elderly Residents of Selected Old Age Homes in Kolkata: A Cross-Sectional Study

**DOI:** 10.7759/cureus.112330

**Published:** 2026-07-09

**Authors:** Nivedita Bharti, Aditi A Jaiswal, Debarati Routh, Subhajoy Maitra, Vikram Pratap

**Affiliations:** 1 Community Medicine, Rajendra Institute of Medical Sciences (RIMS), Ranchi, IND; 2 Community and Family Medicine, Ram Krishna Dharmarth Foundation (RKDF) Medical College, Bhopal, IND; 3 Critical Care Medicine, Rabindranath Tagore International Institute of Cardiac Sciences, Kolkata, IND; 4 Community Medicine, Jagannath Gupta Institute of Medical Sciences and Hospital (JIMSH), Kolkata, IND; 5 Paediatrics and Child Health, Gautam Medical College and Hospital, Ranchi, IND

**Keywords:** elderly, functional status, healthy ageing, old age homes, psychological distress, well-being

## Abstract

Background: Population ageing has emerged as a major public health challenge globally and in India. Elderly individuals residing in old age homes often face physical, social, and psychological challenges that may adversely affect their well-being. This study aimed to assess the level of well-being and identify its determinants among elderly residents of selected old age homes in Kolkata.

Methods:A cross-sectional study was conducted among 273 elderly individuals aged ≥60 years residing in four selected old age homes in Kolkata, West Bengal, from November 2020 to October 2022. Data were collected through face-to-face interviews using a predesigned and pretested schedule. Well-being was assessed using the World Health Organization-Five Well-Being Index (WHO-5), functional status using the Katz Activities of Daily Living (ADL) scale, and psychological distress using the Patient Health Questionnaire-4 (PHQ-4). Factors associated with well-being were examined using multivariable logistic regression.

Results:The mean age of the participants was 74.1 ± 7.8 years, and 142 (52.1%) were male participants. The mean WHO-5 score was 13.32 ± 3.72, with 152 (55.7%) of participants demonstrating good well-being. Chronic morbidity was present in 255 (93.5%) of participants, while 253 (92.7%) experienced some degree of psychological distress. Well-being was negatively correlated with age (rho = -0.311, p < 0.001) and psychological distress (rho = -0.242, p < 0.001), and positively correlated with functional status (rho = 0.339, p < 0.001). In multivariable analysis, nuclear family background (adjusted odds ratio (AOR): 1.9; 95% confidence interval (CI): 1.04-3.75), married status (AOR: 2.8; 95% CI: 1.35-6.11), previous government employment (AOR: 3.1; 95% CI: 1.29-7.31), voluntary admission to the old age home (AOR: 3.6; 95% CI: 1.82-7.39), duration of stay ≤2 years (AOR: 4.8; 95% CI: 1.19-19.8), and absence of psychological distress (AOR: 3.0; 95% CI: 1.29-7.31) were independently associated with good well-being.

Conclusion:More than half of the elderly residents demonstrated good well-being, although psychological distress was highly prevalent. Functional independence, psychosocial factors, and psychological health were important determinants of well-being. Strategies aimed at strengthening mental health support, maintaining functional ability, and fostering supportive institutional environments may help improve well-being among institutionalized elderly populations.

## Introduction

Population ageing is one of the most significant demographic transitions of the 21st century. Advances in healthcare, improved living conditions, and declining fertility rates have led to a substantial increase in life expectancy and a growing proportion of older adults worldwide. The United Nations has estimated that by 2050, one in every six people globally will be aged 65 years or older, compared to one in 11 in 2019 [1]. Simultaneously, India is experiencing a rapid demographic shift towards an ageing population, with older adults anticipated to constitute nearly one-fifth of the country's population by 2050 [2]. As longevity increases, older individuals face a higher risk of chronic non-communicable diseases, functional impairments, social isolation, and psychological challenges that may adversely affect their quality of life and overall well-being [3]. Consequently, promoting healthy ageing and improving the well-being of older adults have emerged as major global public health priorities, exemplified by the United Nations Decade of Healthy Ageing (2021-2030) [4].

Well-being consists of physical, psychological, and social dimensions of health and is an important indicator of healthy ageing. Contemporary geriatric care increasingly focuses on enhancing well-being and quality of life, with the WHO-5 Well-Being Index serving as a widely validated measure of positive mental well-being [5,6].

Rapid urbanization, migration of younger family members, increasing workforce mobility, and the gradual shift from joint to nuclear family systems have contributed to the growing institutionalization of older adults in India. Although old age homes provide accommodation and basic healthcare support, residents often experience loneliness, separation from family, loss of social roles, dependency, and reduced social interactions, which may adversely affect their well-being [7,8].

Institutionalized older adults represent a vulnerable and rapidly growing segment of the elderly population, facing unique psychosocial, health, and environmental challenges that differ from those of community-dwelling older adults. Previous studies have identified age, gender, marital status, social support, functional dependency, chronic morbidities, and economic status as important determinants of well-being among older adults; however, these findings have varied across socio-cultural settings [9-13]. Evidence on the determinants of well-being among institutionalized elderly in eastern India, particularly West Bengal, remains limited, with most studies focusing primarily on morbidity patterns or quality of life [14,15]. Understanding these determinants is essential for designing interventions that promote healthy ageing and improve quality of life among elderly residents of old age homes. Therefore, the present study aimed to assess the level of well-being and identify its determinants among elderly residents of selected old age homes in Kolkata.

## Materials and methods

A cross-sectional institution-based study was conducted among 273 elderly individuals aged ≥60 years residing for at least six months in four selected old age homes in Kolkata, West Bengal, between November 2020 and October 2022. Participants who were seriously ill, bedridden, had severe audio-visual disability, or were unable to respond to the interview were excluded from the study.

Sampling

Old age homes were selected by convenience, based on permission and feasibility. Following protocol development, the study tool was piloted in a similar population elsewhere. Eligible residents were identified from institutional registers, and all consenting individuals were included using the census method, resulting in 273 participants. Participants who could not be interviewed during the scheduled visit were approached on subsequent visits according to their availability, ensuring complete data collection with no missing data.

Data collection, study tools, and parameters used

Data were collected through face-to-face interviews using a predesigned, pretested, interviewer-administered schedule that captured socio-demographic characteristics, behavioural profile, family support, duration of stay in the old age home, and morbidity profile. Well-being was assessed using the World Health Organization-Five Well-Being Index (WHO-5) [16], functional status using the Katz Activities of Daily Living (ADL) scale [17], and psychological distress using the Patient Health Questionnaire-4 (PHQ-4) [18]. Relevant clinical information was supplemented by review of available medical records. The WHO-5 score ranges from 0 to 25, with higher scores indicating better well-being. Participants with a WHO-5 raw score <13 were classified as having poor well-being, while those with a score ≥13 were classified as having good well-being, in accordance with the recommended WHO-5 screening threshold and standard interpretation of the instrument. Well-being status was considered the primary outcome variable, while socio-demographic, behavioural, functional, psychological, and morbidity-related characteristics were treated as explanatory variables.

Data analysis

Data were analysed using IBM SPSS Statistics version 25 (IBM Corp., Armonk, NY). Descriptive statistics were expressed as mean ± SD, median (IQR), frequencies, and percentages. Associations between well-being and explanatory variables were assessed using the chi-square test and Spearman's correlation.

Variables demonstrating a p-value <0.20 in the univariate (bivariate) analysis, along with clinically relevant variables identified from the literature, were considered for inclusion in the multivariable binary logistic regression model. Adjusted odds ratios (AORs) with 95% confidence intervals (CIs) were reported. Multicollinearity among the independent variables was assessed using the variance inflation factor (VIF), with all VIF values <5 indicating no significant multicollinearity. The goodness-of-fit of the final model was evaluated using the Hosmer-Lemeshow test, which indicated an adequate model fit (p>0.05). A p-value <0.05 was considered statistically significant.

Ethical approval

The study protocol received approval from the Institutional Ethics Committee vide approval number AIIHPH/PSM/Protocol/2020-23/015 dated October 2020. A Subject Information Sheet was provided to each participant in their local language, and written informed consent was obtained. Participant anonymity was preserved throughout the study in accordance with the Declaration of Helsinki.

## Results

Socio-demographic characteristics of study participants

The mean age of the study participants was 74.1 ± 7.8 years. The highest proportion was noted in the age group of 70-79 years. Near-equal distribution was present between the genders, with a slightly higher male predominance being noted at 52.1%. Nearly half of the study participants had been widowed. Out of the total, 95 (34.7%) participants were graduate, and the majority came from joint families (163, 59.7%).

Regarding previous occupation, 80 (29.3%) participants had worked in government service, 84 (30.8%) in non-government occupations, and 109 (39.9%) were unemployed/homemakers. The duration of stay in the old age homes was 0-2 years for 64 (23.4%) participants, 3-5 years for 101 (37.0%), and more than six years for 108 (39.6%). A total of 121 (44.3%) participants had entered the old age home voluntarily, whereas 152 (55.7%) had been admitted at the request of family members or due to other circumstances.

Health, functional, and psychological profile

**Figure 1:**
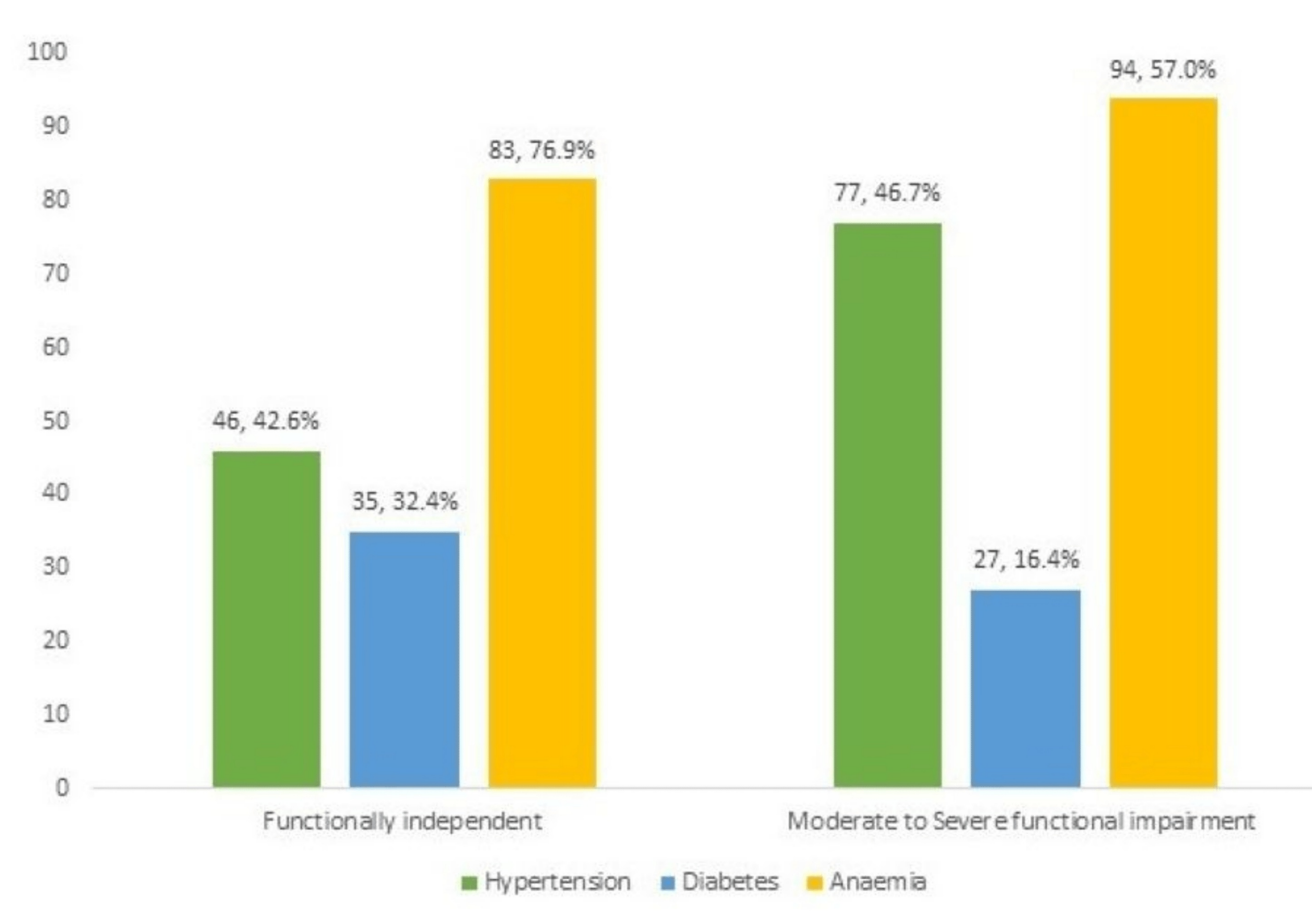
Bar chart showing distribution of study participants across Katz-ADL and presence of comorbidities (N = 273) ADL, activities of daily living.

Chronic morbidity was present among 255 (93.5%) participants, with 110 (40.3%) having multimorbidity. Anaemia was the most common morbidity, followed by hypertension (63.0%) and diabetes mellitus (43.2%). Functional assessment using the Katz ADL scale showed that 109 (39.9%) participants were functionally independent, 155 (56.8%) had moderate impairment, and 9 (3.3%) had severe impairment, with a mean ADL score of 5.07 ± 1.01. Anaemia was highly prevalent among both functionally independent (76.9%) and impaired participants (57.0%), while hypertension was more common among those with functional impairment and diabetes among those who were functionally independent (Figure 1). Psychological distress assessed using the PHQ-4 revealed that only 20 (7.3%) participants had normal scores, whereas 115 (42.1%), 107 (39.2%), and 31 (11.4%) had mild, moderate, and severe distress, respectively. Overall, 92.7% of the participants experienced some degree of psychological distress.

Well-being status

The mean WHO-5 well-being score among the study participants was 13.32 ± 3.72, with a median (IQR) of 13 (11-15) and a range of 4-25. Based on the WHO-5 score, 152 (55.7%) participants had good well-being (score ≥13), while 121 (44.3%) had poor well-being (score <13). More female participants than male participants were found to have poor well-being (57.1% vs. 42.9%).

Correlation analysis

A statistically significant negative correlation was observed between well-being score and age (Spearman's rho = -0.311, p < 0.001), indicating that well-being decreased with advancing age. Well-being showed a significant positive correlation with functional status as measured by the Katz ADL score (Spearman's rho = 0.339, p < 0.001), suggesting better well-being among participants with greater functional independence. Conversely, a significant negative correlation was observed between well-being and psychological distress measured using the PHQ-4 score (Spearman's rho = -0.242, p < 0.001), indicating lower well-being among participants experiencing higher levels of psychological distress.

Factors associated with well-being

Bivariate analysis demonstrated significant associations between good well-being and age group (χ² = 12.708, p = 0.005), family type (χ² = 5.871, p = 0.015), educational status (χ² = 5.430, p = 0.020), marital status (χ² = 5.816, p = 0.016), past occupation (χ² = 8.461, p = 0.015), duration of stay in the old age home (χ² = 28.163, p < 0.001), arrival mode (χ² = 9.595, p = 0.002), and psychological distress (χ² = 10.302, p = 0.001). Good well-being was more frequently observed among younger participants (60-75 years), those from nuclear families, graduates, married individuals, previously employed participants, residents with shorter duration of stay, those who entered the institution voluntarily, and participants without psychological distress. No significant association was observed with gender (χ² = 0.223, p = 0.637) or caste (χ² = 2.762, p = 0.098) (Table 1).

**Table 1:** Association of socio-demographic, behavioral, and clinical characteristics with well-being status among participants (N = 273) *Others: separated, divorced, widow, never married.

Variable	Category	Good well-being, n (%)	Poor well-being, n (%)	χ² (df)	p-Value
Age group	60-75 years (n = 166)	108 (65.1)	58 (34.9)	12.708 (1)	0.005
	>75 years (n = 107)	44 (41.1)	63 (58.9)		
Gender	Male (n = 142)	81 (57.0)	61 (43.0)	0.223 (1)	0.637
	Female (n = 131)	71 (54.2)	60 (45.8)		
Caste	Others (n = 208)	110 (52.9)	98 (47.1)	2.762 (1)	0.098
	OBC/SC/ST (n = 65)	42 (64.6)	23 (35.4)		
Family type	Joint (n = 163)	81 (49.7)	82 (50.3)	5.871 (1)	0.015
	Nuclear (n = 110)	71 (64.5)	39 (35.5)		
Educational status	≤Higher secondary (n = 150)	74 (49.3)	76 (50.7)	5.430 (1)	0.020
	Graduate and above (n = 123)	78 (63.4)	45 (36.6)		
Marital status	Married (n = 74)	50 (67.6)	24 (32.4)	5.816 (1)	0.016
	Others* (n = 199)	102 (51.3)	97 (48.7)		
Past occupation	Government job (n = 80)	50 (62.5)	30 (37.5)	8.461 (2)	0.015
	Non-government job (n = 84)	53 (63.1)	31 (36.9)		
	Unemployed/Homemaker (n = 109)	49 (44.9)	60 (55.1)		
Duration of stay	0-2 years (n = 64)	49 (76.5)	15 (23.5)	28.163 (2)	<0.001
	3-5 years (n = 101)	62 (61.4)	39 (38.6)		
	>6 years (n = 108)	41 (38.0)	67 (62.0)		
Arrival mode	Self (n = 121)	80 (66.1)	41 (33.9)	9.595 (1)	0.002
	Family-requested/forced (n = 152)	72 (47.4)	80 (52.6)		
Psychological distress	Normal (n = 20)	18 (90.0)	2 (10.0)	10.302 (1)	0.001
	Mild/Moderate/Severe (n = 253)	134 (53.0)	119 (47.0)		

After adjustment for potential confounders in multivariable logistic regression analysis, participants from nuclear families (AOR: 1.9; 95% CI: 1.04-3.75), those with a history of government employment (AOR: 3.1; 95% CI: 1.29-7.31), married individuals (AOR: 2.8; 95% CI: 1.35-6.11), participants who had voluntarily entered the old age home (AOR: 3.6; 95% CI: 1.82-7.39), and those residing in the institution for ≤2 years (AOR: 4.8; 95% CI: 1.19-19.8) were significantly more likely to have good well-being. Absence of psychological distress was also independently associated with good well-being (AOR: 3.0; 95% CI: 1.29-7.31) (Table 2).

**Table 2:** Logistic regression -- Factors associated with good well-being among study participants (N = 273) AOR, adjusted odds ratio; CI, confidence interval. *Others: separated, divorced, widow, Never married.

Variable	Category	Bivariate p-value	AOR (95% CI)	Adjusted p-value
Age group	60-75 years vs >75 years	0.005	1.1 (0.54-2.46)	0.714
Past family type	Nuclear vs Joint	0.015	1.9 (1.04-3.75)	0.035
Educational status	Graduate and above vs ≤Higher secondary	0.020	2.5 (0.45-14.0)	0.287
Marital status	Married vs Others*	0.016	2.8 (1.35-6.11)	0.006
Past occupation	Government job vs Unemployed/Homemaker	0.015	3.1 (1.29-7.31)	0.011
Duration of stay	0-2 years vs >6 years	<0.001	4.8 (1.19-19.8)	0.028
Arrival mode	Self vs Family-requested/forced	0.002	3.6 (1.82-7.39)	<0.001
Psychological distress	Normal vs Distressed	<0.001	3.0 (1.29-7.31)	0.011

## Discussion

The present study assessed well-being and its determinants among elderly residents of selected old age homes in Kolkata. More than half of the participants (55.7%) reported good well-being, while 44.3% had poor well-being. Well-being was significantly associated with age, functional status, psychological distress, marital status, family structure, duration of stay in the institution, previous occupation, and mode of admission to the old age home.

In the present study, 55.7% of participants demonstrated good well-being. This finding is comparable to studies conducted among institutionalized elderly in India, which have reported moderate levels of psychological well-being despite the challenges associated with ageing and institutional living. However, the substantial proportion of participants with poor well-being highlights the continuing psychosocial vulnerabilities faced by elderly residents of old age homes. Social isolation, reduced family interactions, and loss of social roles have been identified as important contributors to compromised well-being among institutionalized older adults [19,20].

Advancing age was significantly associated with lower well-being scores, with a negative correlation observed between age and WHO-5 scores. Similar findings have been reported among older adults in India and other low- and middle-income countries, where increasing age was associated with poorer psychological well-being and life satisfaction [11]. This may be attributable to increasing functional dependency, multimorbidity, bereavement, and declining social engagement with advancing age.

Functional independence emerged as an important determinant of well-being in the present study. Participants with higher Katz ADL scores demonstrated significantly better well-being, and a positive correlation was observed between functional status and WHO-5 scores. Similar associations have been reported in previous studies, which showed that elderly individuals capable of performing ADLs independently experience greater autonomy, self-esteem, and social participation, thereby enhancing their overall well-being [20,21].

Psychological distress was highly prevalent in the study population, with more than 90% of participants experiencing some degree of distress. Furthermore, psychological distress showed a significant inverse correlation with well-being, and the absence of distress independently predicted good well-being in multivariable analysis. This finding is consistent with previous literature demonstrating that symptoms of anxiety and depression adversely affect life satisfaction, social functioning, and perceived quality of life among older adults [11,19,22]. The strong association observed in the present study underscores the importance of regular mental health screening and psychosocial support services in old age homes.

Marital status was another significant predictor of well-being. Married participants were nearly three times more likely to report good well-being than those who were unmarried, widowed, separated, or divorced. Similar observations have been reported in studies among Indian older adults, where the presence of a spouse was found to provide emotional security, social support, and companionship, thereby protecting against psychological distress and loneliness [11].

Interestingly, participants belonging to nuclear families prior to institutionalization demonstrated better well-being than those from joint families. Although joint family systems are traditionally considered protective in Indian society, recent evidence suggests that the quality of interpersonal relationships may be more important than family structure alone [11]. Elderly individuals from nuclear families may have developed greater self-reliance and adaptability, which could facilitate adjustment to institutional living.

The present study also identified a shorter duration of stay in old age homes as an independent predictor of good well-being. Participants residing in the institution for two years or less had significantly greater odds of reporting good well-being. While evidence regarding this association remains limited, prolonged institutionalization may contribute to emotional detachment, reduced social engagement, and adaptation difficulties. Similar observations have been noted in studies evaluating quality of life among institutionalized elderly populations [20].

Another notable finding was the positive association between voluntary admission to old age homes and well-being. Participants who had entered the institution by their own choice were more likely to report good well-being than those admitted due to family pressure or other circumstances. This finding supports previous evidence highlighting the importance of autonomy, self-determination, and perceived control in promoting psychological well-being among older adults [19].

The present study is strengthened by the use of validated instruments, a comprehensive assessment of socio-demographic, functional, psychological, and health-related factors, and face-to-face data collection over a two-year period. However, the cross-sectional design precludes causal inference, and the findings should be interpreted considering the selected institutional setting, potential interviewer bias, and the possibility of residual confounding.

Overall, the findings emphasize that well-being among institutionalized elderly persons is influenced by a complex interplay of demographic, functional, psychological, and social factors. Interventions focusing on maintenance of functional independence, early identification of psychological distress, strengthening social support systems, and promoting autonomy may substantially improve well-being among residents of old age homes.

## Conclusions

Well-being among elderly residents of old age homes was influenced by a combination of demographic, functional, psychological, and institutional factors. Better well-being was associated with younger age, greater functional independence, lower psychological distress, favourable social support characteristics, and factors reflecting autonomy and adaptation to institutional living. The findings highlight the need for comprehensive geriatric care that incorporates mental health support, maintenance of functional ability, and a supportive institutional environment to promote healthy ageing and enhance well-being among institutionalized elderly individuals.
